# Timing of Denosumab Therapy in Relation to Parathyroidectomy in the Setting of Primary Hyperparathyroidism: Case Report

**DOI:** 10.1155/crie/8562872

**Published:** 2026-05-30

**Authors:** Ryan S. MacLeod, Adriana Ramirez, Barbara Gulanski, Sachin K. Majumdar

**Affiliations:** ^1^ Department of Internal Medicine, Section of Endocrinology, Yale University School of Medicine, 333 Cedar Street, New Haven, 06510, Connecticut, USA, yale.edu; ^2^ Department of Surgery, Section of Endocrine Surgery, Yale University School of Medicine, 800 Howard Avenue, New Haven, 06519, Connecticut, USA, yale.edu

**Keywords:** case report, denosumab, hyperparathyroidism, parathyroidectomy

## Abstract

The optimal timing of denosumab therapy in relation to parathyroid surgery to avoid hypocalcemia and rebound bone resorption is not well described. Herein we report the case of a 62‐year‐old woman with steroid‐induced osteoporosis who received denosumab for several years prior to undergoing parathyroidectomy. She had a history of lupus and required 20–60 mg of daily prednisone. Prior to her presentation at our institution, she was initially treated with alendronate and calcium/vitamin D supplementation. A dual‐energy X‐ray absorptiometry (DXA) scan 4 years after starting prednisone showed stable osteopenia in the lumbar spine and total hip but osteoporosis in the femoral neck with a significant reduction of bone mineral density (BMD) by 15.4% since baseline. Denosumab was initiated by her local endocrinologist for antiresorptive therapy due to declining bone density while on alendronate. She remained on prednisone for three additional years while on denosumab. 3 years of denosumab therapy resulted in stable bone density. Vitamin D levels were appropriate but elevations in PTH and calcium were noted. What was initially thought to be secondary hyperparathyroidism was at this point consistent with primary hyperparathyroidism (PHPT). A 4‐dimensional computed tomography (4D CT) scan of the neck identified a parathyroid adenoma, and she was referred to our institution for parathyroidectomy. The timing of parathyroidectomy in relationship to the next denosumab injection was a concern due to the rebound bone resorption phenomenon and increased risk of vertebral fractures if denosumab is delayed or missed. Conversely, if denosumab is given in the periprocedural period, severe postsurgical hypocalcemia can result if denosumab prevents liberation of calcium from bone. Considering this, parathyroidectomy was performed 6 months after the last denosumab dose, and it was resumed 8 weeks after surgery. 1 month after surgery, calcium was normal, and PTH had recovered to mid‐normal range. Her postoperative course was uncomplicated, and she was able to resume denosumab.

## 1. Introduction

Primary hyperparathyroidism (PHPT) is the most common cause of hypercalcemia and is due to the inappropriate secretion of PTH from one or more parathyroid glands [1]. Most cases are associated with a single adenoma (80%), but multiglandular disease is also common (20%) [1]. PHPT is diagnosed biochemically by hypercalcemia in the presence of elevated or inappropriately normal PTH levels [1, 2]. The differential diagnosis for hypercalcemia starts by delineating PTH dependence. Medications (thiazide diuretics or lithium), interfering substances (biotin in some assays), and genetic conditions (familial hypocalciuric hypercalcemia) can affect PTH action and measurement, which must be ruled out to confirm the diagnosis of PHPT. Surgical management of PHPT offers the possibility of a definitive cure and should be offered to those who meet any one of the following criteria: hypercalcemia consistently greater than 1 mg/dL above normal range; hypercalciuria defined as 24‐h urinary calcium excretion >250 mg/day (women) and >300 mg/day (men); fragility‐related fracture; nephrolithiasis; bone mineral density (BMD) measurement using dual‐energy X‐ray absorptiometry (DXA) with *T*‐score less than or equal to −2.5 at any site; and age less than 50 years [1–3]. For patients that do not meet these criteria or are poor surgical candidates, monitoring of calcium and PTH status in addition to serial DXA measurements and medical management should be considered [1–3]. Data on the usefulness of bisphosphonates in PHPT are limited, but alendronate has been shown to improve lumbar spine BMD in these patients [4–7]. Denosumab is a monoclonal antibody that targets receptor activator of nuclear factor kappa‐B ligand (RANKL) and interferes with osteoclast differentiation and activity. Denosumab was effective in improving BMD at all measured DXA sites in patients with PHPT in the DENOCINA trial [8], and older women with PHPT had greater increases in BMD with denosumab treatment compared to those with primary osteoporosis [9].

Around 1% of all adults in the United States and 3% of those over 50 years of age receive glucocorticoids for allergies, inflammatory conditions, or cancer [10]. Long‐term use of these agents is associated with several side effects, particularly fractures, which are one of the most common and serious preventable adverse events. Risk of fracture increases with the dose, duration, and age at which glucocorticoids are used [10]. Vertebral fractures are the most common, and their risk increases within 3 months of initiating glucocorticoids at doses over 7.5 mg of prednisone equivalents per day and peak at 12 months [10]. Oral bisphosphonates are recommended as first‐line therapy for glucocorticoid‐induced osteoporosis. A review that included 12 randomized trials and involved 1343 participants showed that those who received bisphosphonates had a 43% reduction in vertebral fracture risk compared to those who received supplementation with calcium, vitamin D, or both [11]. A noninferiority trial comparing denosumab and risedronate in patients just starting glucocorticoid therapy and those who had received glucocorticoids long‐term showed the superiority of denosumab regarding increased BMD at the spine and noninferiority with respect to fracture rate [12]. However, given limited safety data, denosumab is not generally recommended as first‐line treatment and is usually used in circumstances where bisphosphonates are contraindicated or have failed [10].

Herein we describe a case of glucocorticoid‐induced osteoporosis initially treated with oral bisphosphonates with progression of disease and eventual switch to denosumab therapy. During her treatment, what was initially diagnosed as secondary hyperparathyroidism was later recognized as consistent with PHPT, and she was referred for surgical management.

The timing of parathyroidectomy in relation to the denosumab injections is an important consideration for multiple reasons. If denosumab is administered in close temporal proximity to parathyroidectomy, the medication can confound interpretation of intraoperative PTH measurements due to denosumab‐induced hyperparathyroidism and/or cause severe postsurgical hypocalcemia if denosumab prevents liberation of calcium from bone [13, 14]. If denosumab dosing is delayed beyond 6 months, it can increase the risk of the rebound resorption phenomenon associated with hypercalcemia and vertebral fracture, especially in patients with PHPT [15, 16]. Our case presentation outlines these considerations for the timing of denosumab in the setting of parathyroidectomy.

## 2. Case Presentation

**Figure 1:**
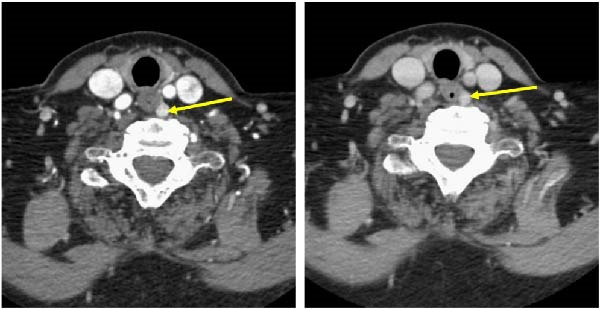
4D Neck CT with and without intravenous contrast. Identification of a hyperenhancing lesion in the neck at the thyroid level, separate from the thyroid gland and abutting the left posterior lateral margin of the esophagus concerning for a parathyroid adenoma.

A 62‐year‐old woman with a history of psoriasis, Grave’s disease status post radioactive iodine ablation, SLE, and angioedema was evaluated by her endocrinologist in the community for thyroid status and bone health. Due to difficulty in controlling her SLE and angioedema symptoms with steroid‐sparing agents, she was treated with glucocorticoid therapy over the past 25 years. She was noted to have mild undertreatment of her hypothyroidism on the tablet form of levothyroxine, so she was started on capsule formulation for presumed malabsorption. She was also noted to have inadequate vitamin D levels (Table 1) and was started on 50,000 IU of ergocalciferol once weekly. Two years prior to presentation to her local endocrinologist, she had a baseline DXA scan with results outlined in Table 2. She was encouraged to repeat a DXA scan but missed several visits with her endocrinologist, and since her rheumatologic concerns persisted, she remained on high‐dose prednisone (40–60 mg daily) and continued high‐dose ergocalciferol. Her rheumatologist started alendronate, 70 mg weekly for bone protection. She had a follow‐up DXA scan 4 years from baseline scan that showed stable osteopenia in the lumbar spine and total hip with *T*‐scores of −1.1 and −2.2, respectively. However, the femoral neck *T*‐score was −3.0, indicating osteoporosis and significant worsening of BMD by 15.4% since baseline (Table 2). Due to worsening bone density on alendronate and continued glucocorticoid therapy, the patient and her local endocrinologist jointly decided to initiate denosumab for antiresorptive therapy. Poor absorption of calcium and vitamin D were thought to contribute to her worsening osteoporosis and presumed secondary hyperparathyroidism—PTH 118.7 pg/mL (15–65 pg/mL) and calcium 9.8 mg/dL (8.8–10.2 mg/dL) (Table 1). Despite remaining on 50,000 IU of ergocalciferol weekly for vitamin D replacement, she only had modest improvement in her vitamin D levels (Table 1). Her prior history of poor absorption of levothyroxine combined with this vitamin D data suggested a general enteral malabsorption of vitamins, nutrients, and medications. She remained on prednisone for 3 additional years while on denosumab. Due to issues with insurance approval and prior authorization documentation for denosumab injections, she frequently had delays in denosumab administration, with most injections occurring every 7–8 months. A follow‐up DXA scan after this period showed lumbar spine *T*‐score of −0.9, total hip *T*‐score of −2.0, and femoral neck *T*‐score of −2.8 with all sites showing nonsignificant increases in BMD, indicating stabilization of her bone loss. Routine labs showed normalization of serum vitamin D 25‐OH levels at 40.7 ng/mL with continued PTH elevations (between 69–178 pg/mL) and intermittent hypercalcemia (range 10.0–11.4 mg/dL) as outlined in Table 3. At that point, it was determined that what was initially thought to be secondary hyperparathyroidism was now consistent with PHPT. A 4‐dimensional computed tomography (4D CT) scan of the neck identified a parathyroid adenoma (Figure 1), and she was referred for parathyroidectomy.

**Table 1:** Timeline of biochemical evaluation for calcium balance.

Assay	Initial presentation	2 years after presentation	6 years after presentation	6.5 years after presentation
Vitamin‐D 25‐OH (30–100 ng/mL)	23.8 ng/mL	33.1 ng/mL	23.4 ng/mL	46.9 ng/mL
Calcium (8.5–10.1 mg/dL)	9.0 mg/dL	9.8 mg/dL	10.0 mg/dL	10.1 mg/dL
Intact PTH (11.7–79.5 pg/mL)	–	–	118.7 pg/mL	68.7 pg/mL
Random urine calcium (mg/dL)	–	–	<2.0 mg/dL	9.8 mg/dL

*Note:* Vitamin‐D 25‐OH, serum calcium, intact PTH, and random urine calcium measurements from patient during endocrinology clinic visits starting with initial presentation to endocrinology. Of note, patient started alendronate in between the first two visits and started on denosumab after the second visit.

**Table 2:** Serial dual‐energy X‐ray absorptiometry measurements.

DXA site	Baseline *T*‐score	4 years after baseline *T*‐score (% change from prior)	7 years after baseline *T*‐score (% change from prior)	9 years after baseline *T*‐score (% change from prior)
Lumbar spine (L1–L4)	−1.2	−1.1 (+1.2%)	−0.9 (+2.0%)	0.1 (+12.3%^a^)
Femoral neck	−2.2	−3.0 (−15.4%^a^)	−2.8 (+4.8%)	−2.7 (+2.7%)
Total hip	−2.0	−2.2 (−2.3%)	−2.0 (+3.5%)	−1.8 (+2.8%)
Distal 1/3 radius	–	–	–	−2.5

*Note*: Lumbar spine, femoral neck, total hip, and distal 1/3 radius measurements of bone mineral density with reported *T*‐score values and percent change from prior measurements. Of note, alendronate was initiated between the first two DXA measurements, and denosumab was initiated and continued from the second DXA measurement onward.

^a^Indicating significant change using DXA machine’s least significant change values (Hologic Horizon A, Software Version 13.6.1.3).

**Table 3:** Confirmation of diagnosis for primary hyperparathyroidism.

Assay	2 months prior to surgery	1.5 months prior to surgery
Vitamin‐D 25‐OH (30–100 ng/mL)	45.2 ng/mL	—
Calcium (8.5–10.1 mg/dL)	11.4 mg/dL	10.6 mg/dL
Intact PTH (15–65 pg/mL)	69.2 pg/mL	79.7 pg/mL
24‐h urine calcium (mg/24 h)	–	326 mg/24 h
24‐h urine creatinine (g/24 h)	–	1.05 g/24 h
24‐h urine volume (L/24 h)	–	3.5 L/24 h

*Note*: Vitamin‐D 25‐OH, serum calcium, intact PTH, and 24‐h urine collection measurements from patient evaluation for suspected primary hyperparathyroidism after years of presumed secondary HPT diagnosis.

The timing of parathyroidectomy in relation to the next denosumab dose is important due to the rebound bone resorption phenomenon and increased risk of vertebral fractures after delayed or missed denosumab doses [13]. Conversely, if denosumab is given in close temporal proximity to parathyroidectomy, it can interfere with intraoperative PTH measurements, and severe hypocalcemia can result if potent antiresorptive therapy prevents liberation of calcium from bone [14, 17]. Due to extenuating circumstances, the patient was unable to time her denosumab injection in relation to parathyroidectomy. Her parathyroid surgery was performed 6 months after the last denosumab dose, and the next dose was given 2 months after surgery. She had left superior and right superior parathyroid gland removal, with surgical pathology indicating enlarged and cellular parathyroid gland tissue. The left excised gland measured 0.9 cm and was 152 mg, and the right gland was 0.6 cm and 100 mg. Initial postoperative PTH was low at 14.3 pg/mL (15–65 pg/mL) with calcium remaining elevated at 10.7 mg/dL (8.8–10.2 mg/dL). One month after surgery, calcium was in normal range at 9.8 mg/dL (8.8–10.2 mg/dL) and PTH had also normalized to 58.5 pg/mL (15–65 pg/mL). She experienced no hypocalcemia symptoms or vertebral fractures since her surgery or with subsequent denosumab injections.

## 3. Discussion

Denosumab is a potent monoclonal antibody targeting RANKL and prevents binding to its receptor, similar to the action of the endogenous soluble decoy receptor osteoprotegerin (OPG). The effect of this treatment reduces new osteoclast formation and inhibits the function and survival of mature osteoclasts on the bone surface [14]. This prevents resorption of bone and can result in potential severe hypocalcemia and elevations in parathyroid hormone. The degree of PTH elevation is highly variable in reported cases, and it is difficult to predict what factors or comorbid conditions contribute to this elevation [14, 18–20]. This makes intraoperative measurements of PTH difficult to interpret with patients on denosumab therapy undergoing parathyroidectomy [14].

In our patient the initial treatment of glucocorticoid‐induced osteoporosis and suspected secondary HPT was complicated by the later emergence and manifestations of PHPT, which had been managed medically with denosumab for several years. After establishing the diagnosis of PHPT, she was referred for parathyroidectomy for definitive management. There are no studies specifically designed to address the optimal timing of denosumab dosing in relation to parathyroidectomy, and recommendations found in the literature are based solely on observational data and theoretical mechanisms of effects on bone and calcium balance of antiresorptive therapies and parathyroidectomy. Therefore, additional cases of this clinical dilemma are important to distribute to the medical community to address this knowledge gap.

It is interesting that this patient’s PHPT did not cause hypercalcemia initially, although denosumab’s potent inhibition of bone resorption is used as primary medical management of PHPT‐induced hypercalcemia in cases where surgery is refused or contraindicated [1, 9, 21–23]. However, elevated PTH can also induce 1‐alpha hydroxylase activity in the kidney, leading to increased calcitriol and subsequently higher calcium absorption in the gastrointestinal tract. In addition, this patient had several denosumab doses delayed past the recommended interval of 6 months which could have also been associated with elevated serum calcium levels secondary to the rebound resorption phenomenon with denosumab withdrawal [13, 15]. While clinical trials show denosumab continues to have both clinical and biochemical effects for up to 8 months [24–26], there may be individual differences in severity and onset of this rebound resorption phenomenon, and vertebral fractures have been reported as early as 8 months after denosumab injection [27–31]. Although this patient had no poor outcomes from her delayed denosumab in relation to her parathyroidectomy, there is no consensus recommendation regarding the correct timing of surgery in relation to denosumab treatment. One strategy is to time parathyroidectomy before the next dose of denosumab, perhaps towards the end of the 6‐month treatment cycle [14]. This strategy takes advantage of declining monoclonal antibody levels and antiresorptive effects to reduce the risk of severe hypocalcemia due to the inability to liberate skeletal calcium stores following parathyroidectomy without increasing the risk of rebound bone resorption and vertebral compression fractures associated with full denosumab withdrawal [13, 15, 32]. In our case, surgery was performed 6 months after the last denosumab dose, and the next dose was given 2 months after surgery without complications of hyper or hypocalcemia. This is in line with data showing denosumab’s continued effects up to 8 months postadministration [24–26].

Our institution has adopted a general strategy to time surgery between 4 and 5 months after the most recent denosumab dose. Following surgery, we advise patients to wait a minimum of 4 weeks before receiving the next denosumab dose. However, this strategy is based on minimal data and mostly relies on anecdotal experience. This case highlights the need to design studies to specifically address the optimal timing of parathyroidectomy in relation to denosumab administration.

## Author Contributions

The manuscript of this case report was written primarily by Ryan S. MacLeod, and all authors provided clinical context to the case presented. Adriana Ramirez assisted with surgical and anatomical descriptions, while Barbara Gulanski and Sachin K. Majumdar were involved in endocrine neoplasia and bone metabolism discussions of the case.

## Funding

No funding was received for this manuscript.

## Disclosure

All authors have read and approved the final version of the manuscript. This case was previously presented as a poster presentation at ENDO 2025 in San Francisco, California in June 2025 [33]. Ryan S. MacLeod had full access to all of the data in this study and takes complete responsibility for the integrity of the data and the accuracy of the data analysis.

## Conflicts of Interest

The authors declare no conflicts of interest.

## Data Availability

The data that support the findings of this study are available upon request from the corresponding author. The data are not publicly available due to privacy or ethical restrictions.
